# Hippocampal Functional Connectivity and Cognitive Stability in Parasellar Meningiomas (Studied by the “Virtually Implanted Electrode” Method)

**DOI:** 10.17691/stm2024.16.6.01

**Published:** 2024-12-27

**Authors:** E.M. Savkina, A.V. Vartanov, M.Yu. Kaverina, A.Yu. Kuleva, M.V. Galkin, O.A. Krotkova

**Affiliations:** PhD Student, Faculty of Psychology; Lomonosov Moscow State University, 1 Leninskie Gory, Moscow, 119991, Russia; PhD, Senior Researcher, Faculty of Psychology; Lomonosov Moscow State University, 1 Leninskie Gory, Moscow, 119991, Russia; Junior Researcher, Department of Neurorehabilitation; N.N. Burdenko National Medical Research Center of Neurosurgery, Ministry of Health of the Russian Federation, 16, 4^th^ Tverskaya-Yamskaya St., Moscow, 125047, Russia; Junior Researcher, Laboratory of General and Clinical Neurophysiology; Institute of Higher Nervous Activity and Neurophysiology, Russian Academy of Sciences, 5A Butlerova St., Moscow, 117485, Russia; MD, PhD, Researcher, Department of Radiotherapy; N.N. Burdenko National Medical Research Center of Neurosurgery, Ministry of Health of the Russian Federation, 16, 4^th^ Tverskaya-Yamskaya St., Moscow, 125047, Russia; PhD, Senior Researcher, Department of Neurorehabilitation; N.N. Burdenko National Medical Research Center of Neurosurgery, Ministry of Health of the Russian Federation, 16, 4^th^ Tverskaya-Yamskaya St., Moscow, 125047, Russia

**Keywords:** hippocampus, cognitive processes, brain network connectivity, the default mode network, “Virtually Implanted Electrode”, method

## Abstract

Adaptive reactions of the brain ensure cognitive stability of the individual in a fairly wide range of pathological impacts, but the mechanisms for the implementation of such compensatory changes have been poorly studied.

**The aim of the study** was to describe changes in the functional connections of the hippocampus subject to a mild unilateral compression in a sample of patients with parasellar meningiomas.

**Materials and Methods:**

A homogeneous sample of 28 patients with parasellar meningiomas adjacent to the hippocampus has been studied. In 16 patients, the tumor was diagnosed on the left side, in 12 patients on the right side. These two groups were comparable in terms of tumor morphometric characteristics and the degree of hemispheric compression. The control group consisted of 31 healthy subjects. All three groups were comparable in age and gender. The “Virtually Implanted Electrode” method was used to describe changes in brain network connectivity. The method allows for the reconstruction of electrical activity in any brain voxel based on its coordinates relative to scalp electrodes. To describe the functional connectivity of the brain, correlation coefficients between all pairs of the selected areas of interest were sequentially calculated.

**Results:**

The comparison of functional connections of the hippocampus in clinical groups and in a group of healthy participants made it possible to identify the following types of dynamics. The first type involves strong and stable hippocampal connections that have not been affected by the pathological process. These are the connections of the hippocampus with the deep stem formations, amygdala, putamen, globus pallidus, and insula. The second type in the clinical groups is characterized by weakening of functional connections of the hippocampus with the structures that transform afferent information flows. Hypothetically, such a weakening could lead to a change in the thresholds of the hippocampal “marking the degree of novelty” of external information flows, being an important way to save individual’s resources. The third type is characterized by enhanced functional connections of the hippocampus with the structures supporting executive functions in clinical groups, which is consistent with the facts of increased voluntariness in the implementation of cognitive actions. Compensatory processes of the brain are not symmetrical. The left and right hippocampi differentially alter functional connectivity under adverse conditions. Restructuring of the interhemispheric interaction may also be considered as a factor ensuring cognitive stability.

**Conclusion:**

Changes in the hippocampal functional connections, identified in the clinical groups by the “Virtual Implanted Electrode” method, can be considered as an adaptive brain reaction aimed at maintaining cognitive stability in parasellar meningiomas.

## Introduction

A network principle of brain implementation of cognitive events [1–4] implies the presence of activity in the spatially separated and spontaneously oscillating cerebral loci. These correlations are considered as a manifestation of self-organization of distributed neuronal elements into a network to process information demanded by the organism at this moment in time. Of special interest are the cerebral networks, which are formed in the resting period (not performing any external cognitive tasks) [5–7]. The default mode networks are believed to provide spontaneous flow of thoughts not connected with concurrent impressions but turned to the previous subject experience [8, 9]. When there appears an external cognitive task, these basic processes are transformed into a functional system of its solution [10–13]. In healthy individuals, the default mode networks are characterized by a relative spatial stability and the time-reproducible structure of the brain loci [8, 14]. The change of the functional connections is registered in aging [15–17] and in various pathological conditions, for example, schizophrenia [18–20], depression [21–23], bipolar disorders [24, 25]. However, the cause-andeffect relationships between the alteration in the brain area connectivity and cognitive processes have not been established up to the end. Little is also known about the mechanisms providing human cognitive stability: the registered changes in speech, perception, memory, and thinking under the condition of a large scope of unfavorable impacts on the brain, despite the forming variability, continue ensuring adaptive behavior of a person in society.

Hippocampus is one of the key structures of the default mode networks [26–29]. Its important role in cognitive processes has never been in doubt [16, 30–33]. Nevertheless, the role of hippocampal functional connections in adaptive alterations has not been actually investigated. Previously [34], we have studied a group of patients with parasellar meningiomas adjacent to the hippocampus compressing it mildly. These extracerebral neoplasms are located at the base of the brain in the immediate vicinity to the medio-basal parts of the left or right temporal lobe. The tumors were shown not to infiltrate the brain substance, i.e. compression of one of the hemispheres occurs without any visible damage to the macrostructures. A slow growth of these neoplasms facilitates the compensatory rearrangements explaining a long-term absence of clinical and neuropsychological symptoms. We believe that the description of functional hippocampal connections in this sample will allow us to identify the mechanisms of compensatory changes involving also the hippocampus.

The functional connections are determined by establishing the degree of the mutual effect of oscillations in any of the nodes of the functioning network on the oscillations in its other nodes. Such correlations are calculated by various methods (fMRI, fNIRS, MEG, EEG), however, there are no uniform, generally accepted standards of recording the network support of cognitive processes [35–39]. The “Virtually Implanted Electrode” method opens quite new opportunities in studying the network connectivity of brain [40–42]. Using scalp EEG, this method enables the reconstruction of electrical activity in any brain voxel with the specified coordinates relative to the scalp electrodes.

The signal from the given area is spatially filtered based on the correlation of signal changes by the leads. The results obtained are interpreted as an electrical activity of the “local field” formed by the virtual electrode “implanted” into the appropriate brain point. This method may be applied to the same EEG data the unlimited number of times testing independently various brain areas. To describe the functional connectivity, the correlation coefficients are calculated successively between all pairs (up to 820 pairs) of the selective zones of interest [42].

**The aim of the study** is to describe the changes in the functional connections of the hippocampus subject to a mild unilateral compression in a sample of patients with parasellar meningiomas.

**The tasks of the investigation:**

1. to describe a network of hippocampal functional connections in the norm using the “Virtually Implanted Electrode” method;
2. to reveal specific features of the functional connections of the hippocampus in patients with its mild unilateral compression;
3. to analyze the role of changes of the hippocampal functional connections in maintaining cognitive stability in patients of the clinical sample.

## Materials and Methods

A homogeneous sample of 28 patients with parasellar meningiomas at the age of 32‒68 years (M±SD — 51.03±13.33) has been studied; women composed 75% of the group. In all cases, the “benign meningioma” diagnosis was established based on the typical clinical and neuroimaging data. 16 patients were diagnosed to have a left-sided tumor location (further in the text the group will be designated as grL), while 12 patients had a right-sided location (grR). The groups were comparable by morphometric tumor characteristics and the degree of the hemispheric compression. None of the patients have previously undergone radiotherapy and neurosurgical interventions.

**Figure 1.**
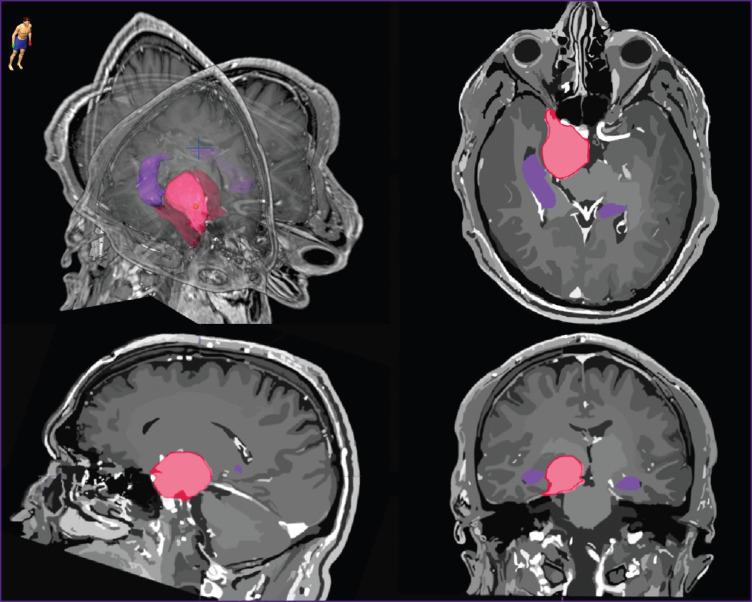
The scheme of tumor location

All patients were performed topometric MRI of the head in the axial projection and the 3D SPGR mode (before and after the introduction of the contrast agent with a 1.0 mm slice thickness) and in the T2 mode (before contrast agent introduction with 2.0 mm slice thickness). To assess more exactly the volume of the tumor and its spatial location, manual segmentation of the tumor and hippocampus was done in the BrainLab iPlan treatment planning system. The hippocampus contouring was done based on the RTOG 0933 protocol and the work of Chera et al. [43] on the axial images successively on each slice using all available modalities. The scheme of location of the tumor and main critical structures is presented in Figure 1. The study was performed before the beginning of radiotherapy at the Department of Radiotherapy of the N.N. Burdenko National Medical Research Center of Neurosurgery (Russia).

The control group consisted of 31 healthy individuals. All three groups were comparable in terms of age and gender. All participants were right-handed.

Patients of the clinical groups (grL and grR) had an active lifestyle, continued working consistent with their age and professional capabilities, came to Moscow by themselves, and underwent the required diagnostic investigations. In addition to the traditional A.R. Luria’s neuropsychological methods, these patients were examined according to the author’s method with a conventional name the “EAM method” (meaning eye-tracking‒attention‒memory). The process of recognition in the EAM method models the involvement of the hippocampus in the mnestic processes [44–48]. In the first point of observation (before the radiotherapy), the test parameters in patients with parasellar meningiomas were within the normal range, which allows us to consider this clinical sample as being in the state of cognitive stability before the beginning of radiotherapy [49].

**Figure 2.**
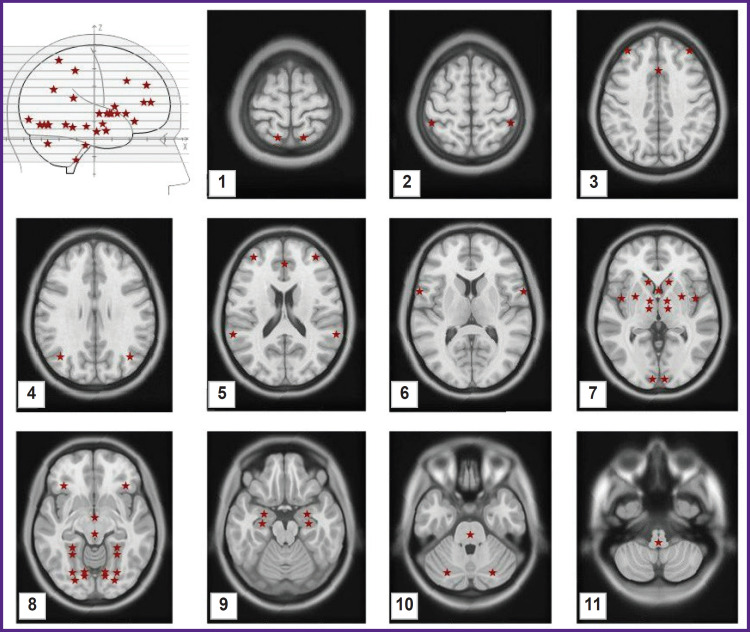
Positions of points of interest Brain images are taken from the MNI atlas; the points of interest (red asterisks) are drawn by the authors: *1* — parietal cortex BA7; *2* — supramarginal gyrus BA40; *3* — dorsomedial prefrontal cortex BA9, middle part of the callosal gyrus; *4* — angular gyrus ВА39; *5* — middle frontal gyrus ВА10, anterior part of the callosal gyrus, superior frontal gyrus ВА22; *6* — inferior frontal gyrus BA44; *7* — head of the caudate nucleus, ventral striatum ВА25, putamen, globus pallidus, insula (insular cortex) ВА13, thalamus, visual field V1 BA17; *8* — orbitofrontal cortex BA47, hypothalamus, mesencephalon, parahippocampal cortex PHC2, parahippocampal cortex PHC1, visual field V4, visual field V3v, visual field VO1, visual field VO2; *9* — amygdala, hippocampus; *10* — medulla oblongata, cerebellum; *11* — brain stem

EEG was recorded in a quiet awake state sitting with the eyes closed. The Neuro-KM encephalograph (Statokin, Russia) with 1000 Hz discretization frequency and a passband from 0.3 to 30.0 Hz was used in our study. Recording was done from 19 leads in compliance with the international 10–20% electrode placement system. The BrainSys software (BrainWin) was used to process the EEG data. The “Virtually Implanted Electrode” method [40–42] was employed to reconstruct the activity in 53 zones of interest (Figure 2) with subsequent calculation of the degree of their functional connectivity.

The study complies with the Declaration of Helsinki (2013) and approved by the local ethics committee of the N.N. Burdenko National Medical Research Center of Neurosurgery (Russia). Written informed consent for participation in the study was obtained from all participants.

### Statistical analysis

The connection strength between the selected loci of interest with the correlation coefficients (modulo) greater than 0.4 was considered significant. Since the correlation coefficients were calculated for the samples of a large size (at digitization frequency of 1 ms, 60,000 cases were compared per 1 min), all sample correlation coefficients were always significantly different by the formal criteria at the level of P=0 (there are no tables of significance for correlation coefficients of large-volume samples), and even corrections for multiple comparisons did not change the fact of overall differences in all coefficients obtained.

This situation seemed incorrect for the informative analysis. In order to reduce the risk of false-negative results, it was decided to evaluate statistically the number of really independent comparisons based on the factor analysis. The number of independent comparisons in this condition may be determined by the number of main components explaining the essential part of data variability. Thus, the criterion for determining statistical significance of differences, considering corrections for multiple comparisons, will be the value of αGWAS=α/ncomponents, where n is the number of the selected components. The value 0.05/n has been used (in our case it was 0.017).

The functional connectivity of the brain structures between the groups was compared using the Student’s t-test on the Statistica software (StatSoft Inc., USA). The Shapiro–Wilk test (W test) was applied to check the normality of distribution. Additionally, the nonparametric U-test was used, the results of selecting significantly different connections by the t- and U-tests coincided.

## Results

To substantiate the homogeneity and comparability of the clinical groups, the volumes of hippocampi were calculated and the tumor topography relative to the main critical structures was determined. An average volume of ipsilateral hippocampus for the whole sample was 3.2 (2.2–4.0) cm^3^, for contralateral — 3.2 (2.4–3.9) cm^3^. There were no statistically significant differences between the grL and grR.

Of 53 zones of interest selected by the “Virtually Implanted Electrode” method, 28 structures have demonstrated significant intrahemispheric correlations of functional activity with the hippocampus (Table 1). The rest 24 selected areas have not demonstrated any significant functional connections with the hippocampus (they are presented in Table 2 for review). The interhemispheric (cross) connections of the hippocampus also have not reached the given level of significance.

**T a b l e 1:** Hippocampal functional connectivity (correlation coefficients) in clinical groups (grL and grR) relative to the control group of healthy participants

Intrahemispheric functional connectivity with selected zones of interest												
for left hippocampus					Selected zones of interest			for right hippocampus				
Norm (1)	grL (2)	grR (3)	p_1–2_	p_1–3_				Norm (1)	grL (2)	grR (3)	p_1–2_	p_1–3_
**0.54**	0.43	0.43	0.1969	0.2280		Brain stem		**0.44**	0.50	0.53	0.5044	0.3825
**0.48**	0.63	0.40	0.0825	0.4311	L	Amygdala	R	**0.49**	0.53	0.49	0.6659	0.9827
**0.77**	0.71	0.71	0.2154	0.2642	L	Putamen	R	**0.75**	0.76	0.78	0.8295	0.4399
**0.75**	0.68	0.73	0.2488	0.8300	L	Globus pallidus	R	**0.76**	0.75	0.73	0.7912	0.4555
**0.70**	0.79	0.70	0.1262	1.0000	L	Insular cortex (insula) BA13	R	**0.68**	0.71	0.73	0.6684	0.4358
**0.55**	**0.20**	***0.29***	**0.0017**	***0.0297***	L	Visual field V4	R	**0.52**	0.40	0.48	0.2103	0.7242
**0.64**	0.50	***0.46***	0.0530	***0.0377***	L	Superior temporal gyrus BA22	R	**0.63**	0.49	***0.41***	0.1425	***0.0279***
**0.66**	**0.36**	0.51	**0.0033**	0.1580	L	Parahippocampal cortex PHC2	R	**0.66**	0.58	0.65	0.2734	0.8710
**0.47**	**0.18**	0.43	**0.0018**	0.6387		Mesencephalon		**0.44**	**0.16**	0.58	**0.0039**	0.0569
**0.55**	**0.33**	0.43	**0.0163**	0.1942		Medulla oblongata		**0.45**	0.42	0.58	0.7173	0.0846
**0.81**	0.76	0.75	0.2854	0.1312	L	Thalamus	R	**0.83**	0.78	**0.75**	0.2961	**0.0113**
0.33	0.40	***0.46***	0.1500	***0.0358***	L	Ventral striatum BA25	R	**0.55**	0.49	**0.40**	0.1500	**0.0020**
0.39	0.33	0.45	0.1314	0.5361		Hypothalamus		0.37	0.25	***0.53***	0.1314	***0.0348***
0.20	**0.49**	**0.59**	**0.0021**	**0.0000**	L	Middle frontal gyrus BA10	R	0.25	**0.43**	**0.52**	**0.0171**	**0.0008**
**0.49**	**0.78**	0.62	**0.0005**	0.1700	L	Inferior frontal gyrus BA44	R	**0.51**	***0.67***	0.64	***0.0271***	0.1748
–0.30	–0.40	–0.41	0.2589	0.3454	L	Angular gyrus BA39	R	–0.39	***–0.57***	**–0.66**	***0.0458***	**0.0021**

* In healthy participants, correlation coefficient values exceeding (modulo) 0.4 are marked in bold. Value differences with statistical significance of 0.017<p<0.05 are shown in semi-bold italics. Value differences with statistical significance of р<0.017 are marked in semi-bold with underscore. The growing interaction in the clinical group in comparison with norm is shown by the red color, while decrease is designated in blue.

**T a b l e 2:** Hippocampal functional connectivity (correlation coefficients) not reaching significance threshold |0.40|

Intrahemispheric functional connectivity with selected zones of interest												
for left hippocampus					Selected zones of interest			for right hippocampus				
Norm (1)	grL(2)	grR (3)	p_1–2_	p_1–3_				Norm (1)	grL (2)	grR (3)	p_1–2_	p_1–3_
–0.14	–0.29	–0.30	0.0848	0.1302	L	Dorsomedial prefrontal cortex BA9	R	–0.14	–0.19	–0.20	0.6084	0.7622
0.18	0.01	0.12	0.0323	0.4421	L	Orbitofrontal cortex BA47	R	0.17	0.02	0.05	0.0900	0.1763
–0.03	0.04	–0.02	0.3322	0.8489	L	Supramarginal gyrus BA40	R	–0.01	0.16	0.08	0.0148	0.2239
–0.06	–0.09	–0.03	0.6170	0.6502	L	Parietal cortex BA7	R	0.02	–0.11	0.00	0.0694	0.8075
0.00	–0.12	–0.11	0.0742	0.1088	L	Visual field V1 BA17	R	–0.02	0.04	0.04	0.3385	0.3593
0.20	–0.12	–0.04	0.0022	0.0267	L	Visual field V3v	R	0.08	0.03	0.13	0.5616	0.6993
0.22	–0.11	–0.04	0.0003	0.0087	L	Visual field VO1	R	0.17	0.07	0.15	0.2400	0.8522
0.12	–0.14	–0.08	0.0017	0.0248	L	Visual field VO2	R	0.05	0.01	0.04	0.5953	0.8858
–0.01	–0.16	–0.13	0.0298	0.1063	L	Parahippocampal cortex PHC1	R	–0.06	–0.01	–0.03	0.4694	0.6585
0.25	0.24	0.31	0.9188	0.4595	L	Head of the caudate nucleus	R	0.33	0.16	0.21	0.0160	0.0790
0.14	–0.16	–0.15	0.0046	0.0076	L	Cerebellum	R	–0.05	–0.04	0.06	0.2707	0.9312
–0.04	0.16	0.18	0.0121	0.0053		Anterior callosal gyrus BA32		0.09	0.11	0.17	0.8099	0.2399
–0.03	0.14	0.11	0.0087	0.0495		Middle callosal gyrus		0.08	0.06	0.10	0.7189	0.8335

In Table 1, we represented the indicators of intrahemispheric functional connectivity of the hippocampus with 28 cerebral structures, 12 of which are paired formations, while 4 are located along the midline. The strength of some connections in the healthy participants and clinical groups was unchangeable (intergroup differences did not reach the level of statistical significance). These were the connections with the brain stem, amygdala, putamen, globus pallidus, and insula.

Other brain loci demonstrated weakening of the functional connections with the hippocampus in the clinical groups relative to the control. For example, in both clinical groups, functional connectivity of the hippocampus and V4 visual field decreased in the left hemisphere (in grL p<0.017; in grR p<0.05). In grL, there was observed a reduced combined functional interconnection of the hippocampus and parahippocampal cortex PHC2 in the left hemisphere (p<0.017), weakened functional connectivity of the mesencephalon with the left and right hippocampi (p<0.017), decreased coherence between the left hippocampus and medulla oblongata (p<0.017). In grR, the functional connectivity of the hippocampus and superior temporal gyrus weakened in the left (p<0.05) and right (p<0.05) hemispheres, and the hippocampus and thalamus in the right hemisphere (p<0.017).

The functional connectivity of the hippocampus and ventral striatum in the control group was asymmetrical, enhanced in the right hemisphere. In grL patients, this tendency remained (the asymmetry was smoothed out but did not reach the level of statistical significance). In grR patients, the connection with the hippocampus enhanced (p<0.05) in the left hemisphere and weakened in the right (p<0.017).

In separate brain structures, strengthening of functional connections with the hippocampus was observed in comparison with the control group. Thus, in both clinical groups, the connectivity of the middle frontal gyrus with the left (p<0.017) and right hippocampus increased (in grL p<0.05; in grR p<0.017). In the right hemisphere, the increase (modulo) of functional connectivity of the angular gyrus with the right hippocampus was noted (in grL p<0.05; in grR p<0.017). In grL, there was increased coherence of the inferior frontal gyrus with the left (p<0.017) and right hippocampi (p<0.05); the connectivity of the right hippocampus with hypothalamus increased in grR (p<0.05).

## Discussion

Let us consider the obtained data in relation to cognitive stability characterizing the clinical sample under study. The results presented in Table 1 allow us to distinguish three types of changes of the hippocampal functional connectome in the clinical groups relative to the control group of healthy participants.

***The first type of connectome changes*** covers strong and stable connections (unaffected by the pathological process). These are connections of the hippocampus with deep stem formations, amygdala, putamen, globus pallidus, and insula. They demonstrated significant correlation coefficients in the control and clinical groups and did not have intergroup differences. Such strength of connections is certainly one of the factors of cognitive stability.

***The second type of connectome changes*** involves the connections characterized by partial weakening in the clinical groups as compared to the norm. Hippocampus is known to function as comparator of impressions [47, 50, 51]. Any impressions of the current experience are compared with the impressions of the previous experience. They may be evaluated as absolutely new, as similar but different in important features, or as completely repeating the previous experience (different in nonessential features). High-resolution multivoxel magnetic resonance spectroscopy provided the opportunity to describe the involvement of hippocampus in these processes [44, 45]. Investigations on model animals also prove the participation of hippocampus in assessing the novelty of the environment [33]. When an animal is moving in the space, the environment is constantly changing. But this context is perceived as constant until a certain threshold is exceeded or some border is crossed. The brain generates adaptive behavior of an individual, which remains constant in a certain range of changes in the input signals from the environment. Hippocampus continuously traces these changes of the context functioning in the mode of continuum. Individuals may detect both similarity and difference of the input information generating an adaptive physiological response. Hippocampal recurrent neural networks are capable of self-organization so that they do not require a priori learning to form a spatiotemporal pattern. The neural loops are constantly active. Information from any source overlaps the current activity and interacts with it in such a way that it may enhance the current trajectory or change it considerably, constantly “running after its own tail”. Hippocampus ensures the continuity of the context due to anatomical neural loops nested within each other, which allows the system to control itself to a larger extent and maintain a reliable circulation of information [33].

However, the continuum of hippocampal impression marking by the degree of their novelty is not constant. It depends on the current physical (physiological) abilities of an individual to assimilate environmental changes. Reduction in functional body reserves results in the increase of threshold recognition of the context and its evaluation as a new one. These changes become especially prominent with age. It has been experimentally established, that at the level of behavioral reactions and by the picture of the multivoxel pattern in the hippocampus for the assessment of the stimuli as similar but not identical, a considerably greater degree of differences between them is required in old age compared to young [52, 53]. Elderly need greater dissimilarity of the input information than young people to make a decision on the difference with the previously encountered analogous information. They are observed to have a shift of information marking towards its summarizing and generalization, whereas in young individuals it is directed towards discrimination of stimuli [45, 52, 54, 55].

In our clinical groups, the adaptive changes in the novelty thresholds for external information flows were likely to occur due to weakened functional connections of the hippocampus with the visual cortex, superior temporal gyrus, thalamus, and other brain loci (see Table 1).

***The third type of connectome changes*** is connected with the increased interaction of the hippocampus with the structures traditionally related to implementation of controlling functions. In the control group, coefficients of functional correlation of this locus do not reach statistical significance, however, in the clinical groups, the connection of the hippocampus with the middle frontal gyrus increases and differs from those in the control group at a high level of statistical significance (p<0.017). The enhancement of functional interaction has occurred bilaterally in both clinical groups (see Table 1). This change indicates the increase of factors of voluntariness and control in realization of behavioral decisions and cognitive functioning. Our patients noted that many tasks, which previously they had solved quite easily, required now special efforts and self-control and only such concentration may help them to fulfil the tasks correctly.

The facts related to the enhancement of the coherent connections of hippocampus with the middle frontal gyrus in pathological conditions were many times reported in the literature. For example, in students with subclinical depression syndrome, the growth of functional connectivity of these structures was registered in restingstate fMRI [23]. The results were compared with the group of healthy students. Besides, a strong positive correlation was observed between the scores of the Beck Depression Inventory and indicators of functional connectivity at the resting state of the hippocampus and middle frontal gyrus. It is likely that boosting the voluntariness factor in cognitive functioning in subclinical depression becomes demanding for the “detachment” from depressive thoughts and emotions, which interfere and hamper the solution of the current life tasks.

The correlation coefficients of functional activity of the hippocampus with the angular gyrus appeared the only negative values in all three groups. It may be proposed that these negative values are caused by the phase shift of the sinusoidal signals.

Investigations using point electrode implantation into definite areas of human hippocampus became possible owing to the rapid development of technologies in neurosurgery. Such interventions are performed by medical indications in patients with pharmacoresistant epilepsy. When perceptive signals reach the hippocampus and trigger the process of recalling, the hippocampal response is first recorded in a highfrequency range (from 50 to 110 HZ) on the intracranial EEG, but after 500 ms, this signal differs depending on successful or unsuccessful recall. If the recall was successful, this gamma-ray burst is followed by a relative power decrease in the alpha-range starting from about 800 ms. It represents a whole cascade of responses in various brain loci: beginning from about 800 ms, it is registered in the medial temporal area, and 900 ms later the posterior parietal cortex is involved. The authors [56] assume that the hippocampal gammaactivity burst corresponds to the moment of comparing perception and memory. This signal is then converted in the temporoparietal regions.

In the group of healthy participants, almost all correlation coefficients were symmetrical (did not differ statistically significantly) for the left and right hippocampi with the selected zones of interest, whereas in the clinical groups this symmetry was broken. The obtained data show that the left and right hippocampi change their connectivity in a different way in response to compression. Evidently, these data will further shed light on specific ways the hemispheres provide cognitive stability, but for the present, we are not ready to present the generalized interpretation of asymmetric reactions of hippocampus on compression.

The structural damages to the left hippocampus mediate behavioral manifestations more explicitly. Laser resection of part of hippocampus in patients with epilepsy results in formation of deficits in the visual fields, and the structural damage to the left hippocampus is followed by more frequent and prominent symptoms than the resection of the right one [57]. In patients with temporal epilepsy, the size of hippocampus always diminished on the side of injury, however the severity of memory defects correlated with the volume of the left hippocampus and did not correlate with the volume of the right [58]. Exposure of the left hippocampus to radiation led to the dose-dependent worsening of delayed verbal reproduction, whereas the right hippocampus did not demonstrate direct correlations of cognitive tests with radiation doses [59, 60]. Specific features of the EEG pattern associated with different lateralization of compression on the hippocampus spoke of the predominance of irritatively epileptiform signs in the left hemisphere [61]. Various degrees of mediation of the cognitive processes by the cellular and neurohumoral constituents of the brain work [62] in combination with the enumerated factors may serve as one of the hypotheses explaining asymmetrical connectivity changes under unilateral action on the hippocampus.

## Conclusion

Generally, a human being retains independent and socially adaptive behavior despite a wide range of pathologic impacts on the brain and variability of cognitive processes associated with them. A network principle of cognitive event implementation suggests that one of the possible mechanisms of such cognitive stability is reorganization of functional brain connections. These processes were studied in the groups with unilateral compression of the hippocampus and adjacent medio-basal areas of the temporal lobe. Meningiomas did not infiltrate the brain, acting without visible injury at the macrostructural level. The change in the functional hippocampal connectivity, detected with the help of the “Virtually Implanted Electrode” method, has demonstrated the following specific features.

Weakening of the functional connections with the structures transforming afferent information flows, which is probably the mechanism of changing the thresholds of “marking the degree of novelty” of information. This change of the thresholds of external information flow novelty may save the individual’s resources.

Boosting of hippocampal functional connectivity with the structures providing control functions may indicate the growth of voluntariness in realization of cognitive events. Transfer of cognitive actions to the voluntary level may be considered as one of the ways of achieving the desired result when difficulties are formed.

The left and right hippocampal hemispheres change their functional connections in different ways in the altering conditions. The compensatory brain processes are not symmetrical. The hypothesis on the mechanism of interhemispheric interaction as one of the essential factors ensuring cognitive stability is increasingly considered in the literature.
